# Predicted indirectly recognizable HLA epitopes scores and clinical outcomes after haploidentical stem cell transplantation in pediatric patients with relapsed neuroblastoma

**DOI:** 10.3389/fimmu.2025.1517387

**Published:** 2025-01-22

**Authors:** Eun Seop Seo, In Hwa Jeong, Hee Young Ju, Ju Kyung Hyun, Ji Won Lee, Keon Hee Yoo, Won Young Heo, Ki Woong Sung, Hee Won Cho, Eun-Suk Kang

**Affiliations:** ^1^ Department of Pediatrics, Samsung Medical Center, Sungkyunkwan University School of Medicine, Seoul, Republic of Korea; ^2^ Department of Digital Health, Samsung Advanced Institute for Health Sciences & Technology (SAIHST), Sungkyunkwan University, Seoul, Republic of Korea; ^3^ Department of Laboratory Medicine and Genetics, Samsung Medical Center, Sungkyunkwan University School of Medicine, Seoul, Republic of Korea; ^4^ Department of Laboratory Medicine, Dong-A University Hospital, Dong-A University College of Medicine, Busan, Republic of Korea

**Keywords:** neuroblastoma, PIRCHE, haploidentical hematopoietic stem cell transplantation, relapse/refractory, HLA mismatch

## Abstract

**Introduction:**

The Predicted Indirectly ReCognizable HLA Epitopes (PIRCHE) model is a recently developed algorithm that predicts indirect T-cell recognition by calculating the number of such epitopes in donor-recipient pairs.

**Methods:**

In this study, the clinical significance of PIRCHE was evaluated in pediatric patients with relapsed/progressed neuroblastoma undergoing haploidentical stem cell transplantation (haplo-SCT).

**Results:**

A higher PIRCHE-I score was associated with faster platelet recovery (*P* = 0.007) and lower incidence of hemorrhagic cystitis (13% vs. 41%, *P* = 0.028) and invasive fungal infections (0% vs. 18%, *P* = 0.045). Additionally, a higher PIRCHE-I score was significantly associated with better overall survival (OS) (HR 0.57, 95% CI 0.34-0.97, *P* = 0.038). A higher PIRCHE-II score was associated with better OS (HR 0.57, 95% CI 0.34-0.94, *P* = 0.028) and reduced progression (HR 0.48, 95% CI 0.30-0.77, *P* = 0.002). When combined, the PIRCHE-I and PIRCHE-II scores demonstrated an even stronger association with improved OS (HR 0.35, 95% CI 0.15-0.82, *P* = 0.016). Multivariable analysis confirmed that a higher combined PIRCHE-I and PIRCHE-II score was independently associated with improved OS (combined PIRCHE score HR 0.22, 95% CI 0.06-0.79, *P* = 0.021), and a higher PIRCHE-II score was significantly associated with reduced progression (HR 0.42, 95% CI 0.25-0.70, *P* < 0.001).

**Conclusion:**

In conclusion, higher PIRCHE-I and PIRCHE-II scores are linked to better survival outcomes and reduced complications in pediatric haplo-SCT neuroblastoma patients. Incorporating PIRCHE scores into donor selection is expected to optimize transplant outcomes.

## Introduction

The degree of human leucocyte antigen (HLA) disparity between donor and recipient is a dominant determinant of clinical outcomes after allogeneic stem cell transplantation (allo-SCT) and is the most important consideration in donor selection. Matching for HLA-A, -B, -C, -DRB1, and -DQB1 alleles (so-called 10/10 match) between the patient and donor is the gold standard for unrelated donor selection (1). Nevertheless, graft-versus-host-disease (GVHD) can occur even in patients transplanted with 10/10 HLA-matched grafts, while it might be absent in certain mismatched transplants (2).

Recently, epitope-based HLA matching algorithms, such as HLAMatchmaker and Predicted Indirectly ReCognizable HLA Epitopes (PIRCHE) have been developed for solid organ and hematopoietic stem cell transplantation (HSCT). HLAMatchmaker evaluates HLA disparity at the eplet level by counting the number of eplet differences between donor and recipient alleles (3–5). Meanwhile, PIRCHE predicts disparity by calculating the number of HLA-derived peptides potentially presented by patient-donor shared HLA molecules, which can trigger an indirect alloreactive response (6). The PIRCHE algorithm, first described in 2013 by Otten et al. in the context of kidney transplantation (7), has since been adapted for stem cell transplant domains (8). The PIRCHE algorithm predicts immunological disparity by calculating the number of HLA-derived peptides potentially presented by shared HLA molecules between donor and recipient. It incorporates several computational steps, including validation of input data, imputation of HLA genotyping data, HLA sequence extrapolation, and peptide binding predictions. The current PIRCHE application, implemented as a web-based tool (www.pirche.com), supports HLA peptide binding prediction using specialized predictors based on the structural properties of the HLA binding groove. The PIRCHE-I and PIRCHE-II scores indicate the number of allogenic HLA peptides presented by HLA class I and class II molecules, respectively (9, 10). Higher PIRCHE scores represent a greater number of potentially immunogenic HLA epitope mismatches, highlighting their theoretical clinical relevance. While the PIRCHE algorithm offers promising insights into immunological compatibility, its application in allo-SCT, particularly in haploidentical stem cell transplantation (haplo-SCT), remains underexplored. In haplo-SCT, characterized by significant donor-recipient HLA disparity, the integration of PIRCHE scoring could provide a more refined method for donor selection and risk stratification, potentially improving outcomes. However, further research is needed to validate the clinical utility of PIRCHE scores in this setting.

In parallel, high-dose chemotherapy and autologous stem cell transplantation (HDCT/auto-SCT) is a crucial part in the treatment of high-risk neuroblastoma (11–13). However, many patients experience relapse/progression, even after tandem HDCT/auto-SCT. The prognosis after relapse/progression has been dismal despite multimodal salvage treatment. In such patients, allo-SCT with graft-versus-tumor (GVT) effect can be a treatment option (14). Haplo-SCT with or without preceding high-dose ^131^I-metaiodobenzylguanidine (HD-MIBG) treatment has been performed as an attempt to enhance the anti-tumor effect in patients with relapsed/progressed neuroblastoma and has shown tolerable toxicity (15, 16).

In this context, our study aims to evaluate the impact of PIRCHE scores on the outcome after haplo-SCT in pediatric patients with relapsed neuroblastoma. We hypothesize that PIRCHE scores could have an impact on outcomes following haplo-SCT, thereby offering guidance for haploidentical donor selection.

## Methods

### Patients and treatment

This study was approved by the Institutional Review Board of Samsung Medical Center (IRB No. SMC 2024-10-055). Patients with neuroblastoma who underwent haplo-SCT from 2012 to 2023 due to relapse/progression after tandem HDCT/auto-SCT were eligible for this study. Salvage chemotherapy was administered to reduce the tumor burden prior to haplo-SCT. The regimen type and duration of chemotherapy depended on tumor response and patient tolerance. Surgical resection and local radiotherapy were performed whenever possible.

At 21 days prior to haplo-SCT, all patients received a single one-hour intravenous infusion of ^131^I-MIBG (18 mCi/kg) with potassium iodide for thyroid protection and intravenous hydration. Patients received reduced-intensity conditioning consisting of cyclophosphamide (60 mg/kg on days -7 and -6), fludarabine (30 mg/m^2^ on days -5 to -1), and rabbit anti-thymocyte globulin [Thymoglobulin, Genzyme; 2.5 mg/kg on days -4 (-3 since 2017) to -1].

Patients received T cell-replete grafts from 2012 to 2016, and α/β T cell-depleted grafts have been used since 2017. While cyclosporine (CSA) and short-course methotrexate (15 mg/m^2^ on day 1 and at 10 mg/m^2^ on days 3 and 6, followed by folic acid rescue) were used to prevent GVHD after haplo-SCT without T cell depletion, only CSA was administered from day -1 in T cell-depleted haplo-SCT. The total duration and tapering schedule of CSA were determined individually according to GVHD severity. Severities of acute and chronic GVHD were recorded based on previously described standard clinical criteria (17).

Antifungal prophylaxis was administered from absolute neutrophil count (ANC) < 0.5×10^9^/L to ANC ≥ 1.0×10^9^/L or during corticosteroid treatment. Acyclovir was used to prevent viral reactivation by day 30, and prophylactic trimethoprim-sulfamethoxazole was administered from neutrophil engraftment until day 180 or immunosuppressant discontinuation. Routine surveillance for cytomegalovirus (CMV), Epstein-Barr virus (EBV), and BK polyomavirus (BKV) reactivation was performed weekly during the first three months post-transplant and monthly thereafter if no viral reactivation occurred. If α/β T cell-depleted grafts were used, rituximab was prophylactically used on day 1. If CMV or EBV load increased, ganciclovir or rituximab was initiated preemptively, respectively.

Tumor response was assessed prior to HD-MIBG treatment, every three months during the first-year post-transplant, every four months during the second year, every six months during the third year, and annually thereafter. International response criteria for neuroblastoma were used to evaluate treatment response (18). When tumors persisted or relapse/progression occurred, salvage treatment was administered upon request.

### HLA/KIR genotyping and donor selection

High-resolution HLA typing was performed with either Sanger sequence-based typing (SBT) (Allele SEQR SBT kit; Atria Genetics, San Francisco, CA; GeneAmp PCR system, Applied Biosystems) or next-generation sequencing (NGS) (AllType™ NGS 11-Loci kit, OneLambda, USA; Illumina NextSeq System). Two-field allele level data for HLA-A, -B, -C, -DRB1, and -DQB1 were acquired from both patients and donors. Killer cell immunoglobulin-like receptor (KIR) genotyping was performed on donor DNA samples using a PCR-based sequence-specific oligonucleotide technique (LIFECODES KIR Typing Kit, Immucor Transplant Diagnostics, Inc., Stanford, CT). Based on HLA type and KIR genotype data, a KIR/HLA-ligand mismatch was defined as incompatibility between the inhibitory donor KIR and recipient HLA class I alleles, as previously described (19). Donor KIR haplotypes were categorized as AA (homozygous for group A KIR haplotypes) or BX (A/B heterozygotes or B/B homozygotes). The KIR B haplotype-defining loci were KIR2DL5, 2DS1, 2DS2, 2DS3, 2DS5, and 3DS1 (20). A haploidentical parent donor with a BX haplotype and/or KIR/HLA-ligand mismatch was preferred.

### Enumeration of PIRCHE scores

PIRCHE scores were acquired through the online PIRCHE matching tool (https://www.pirche.com) by inputting HLA allele data of the patient-donor pairs. PIRCHE scores corresponding to the number of allogeneic HLA peptides capable of inducing an indirect alloreactive response were generated. PIRCHE scores for allogeneic peptides presented by HLA class I molecules (PIRCHE-I) and class II molecules (PIRCHE-II) were computed separately in the GVH direction.

### Statistical analysis

All statistical analyses were performed using R software version 4.4.0 (R Foundation for Statistical Computing, Vienna, Austria). Patient characteristics and transplant-related information were summarized using descriptive statistics. Continuous variables were expressed as medians with ranges, and categorical variables as frequencies and percentages. Non-hematologic outcomes were compared using chi-square tests or Fisher’s exact tests for categorical variables and the Mann-Whitney U test for continuous variables. The PIRCHE scores were dichotomized at their median values to evaluate their association with transplant-related complications, such as acute and chronic graft-versus-host disease (GVHD), hemorrhagic cystitis, and infections. This approach was chosen due to the absence of a universally established cutoff for PIRCHE scores in the existing literature. Using the median as the threshold allowed for a pragmatic and unbiased stratification of the cohort to explore the potential impact of higher versus lower PIRCHE scores on transplant outcomes. Survival outcomes, including overall survival (OS) and progression-free survival (PFS), were analyzed using Cox proportional hazards regression models. Both univariable and multivariable analyses were conducted. Variables with a *P* value < 0.1 in the univariable analysis were included in the multivariable models. PIRCHE-I and PIRCHE-II scores were treated as continuous variables and log-transformed to normalize their distributions. The impact of these scores on survival outcomes was assessed both individually and in combination (PIRCHE-I+II). Hazard ratios (HRs) with 95% confidence intervals (CIs) were reported. All tests were two-sided, and a *P* value < 0.05 was considered statistically significant.

## Results

### Patient and transplant characteristics

Patient characteristics are detailed in **Table 1**. A total of 46 patients were included in this study. The median age at haplo-SCT was 6.6 years (range 3.9 to 12.1). Among patients with known pathology and tumor biology, 7 patients (18%) had tumors with *MYCN* amplification, and 31 patients (67%) had poorly differentiated or undifferentated tumors. The pre-haplo-SCT status was complete response (CR) or very good partial response (VGPR) in 18 patients (39%) and partial response or worse (≤ PR) in 28 patients (61%).

**Table 1:** Patient and transplant characteristics.

Characteristic	N = 46
Age at haplo-SCT	6.58 (3.92,12.06)
Pathology	
Ganglioneuroblastoma/Differentiating	7 (15%)
Poorly differentiated/Undifferentiated	31 (67%)
Unknown	8 (17%)
*MYCN* status	
Not amplified	33 (72%)
Amplified	7 (15%)
Unknown	6 (13%)
Pre-haplo-SCT status	
Complete response/Very Good Partial Response	18 (39%)
Partial Response or worse	28 (61%)
NSE (ng/mL) at relapse	20 (7,373)
CD34+ cells (10^6^ cells/kg)	8.6 (2.2,30.1)
Tαβ cell depletion, n (%)	29 (63%)
KIR Haplotype	
A	16 (35%)
B	30 (65%)
PIRCHE-I	21 (2,58)
PIRCHE-II	32 (4,102)

KIR, killer cell immunoglobulin-like receptor (KIR); NSE, neuron-specific enolase; PIRCHE, predicted indirect recognizable HLA epitope; SCT, stem cell transplantation.

Regarding the transplant characteristics, a median of 8.6 (range 2.2–30.1) × 10^6^ CD34+ cells/kg was transplanted. T cell-replete haplo-SCT was performed in 27% of the patients, with a median of 4.8 (range 1.9–10.5) × 10^8^ CD3+ cells/kg transplanted. α/β T cell-depleted haplo-SCT was performed in 63% of the patients, with a median of 1.9 (range 0.1–22.1) × 10^4^ α/β T cells/kg transplanted. KIR haplotype analysis indicated that 35% of the patients (16 patients) had haplotype A, and 65% (30 patients) had haplotype B. The median PIRCHE-I and PIRCHE-II scores were 21 (range 2–58) and 32 (range 4–102), respectively.

### Association of PIRCHE score with non-survival transplant outcomes

PIRCHE scores were categorized as high and low based on the median value, and transplant outcomes were analyzed according to these stratified score groups (**Table 2**). For PIRCHE-I, despite chimerism at day 30 being slightly higher in the low-score group compared to the high-score group (*P* = 0.044), there was a statistically significant difference in the time to achieve a platelet count of 200,000/µL, with the high-score group recovering faster than the low-score group (*P* = 0.007). Regarding non-hematologic outcomes, the incidence of hemorrhagic cystitis was significantly higher in the low-score group (41%) compared to the high-score group (13%, *P* = 0.028), and invasive fungal infections were more common in the low-score group (18%) compared to none in the high-score group (*P* = 0.045).

**Table 2:** Transplant outcomes stratified PIRCHE score.

Characteristic	High*, N = 24	Low*, N = 22	*P* value
PIRCHE-I	High ( ≥ 21)	Low (< 21)	
Time to ANC 500/µL (days)	10 (8, 22)	11 (7, 15)	0.608
Time to PLT 200,000/µL (days)	17 (15, 180)	21 (16, 180)	0.007
Chimerism at d30	99.7 (65.1, 100)	100 (96.8, 100)	0.044
Acute GVHD, n (%)			0.315
0	13 (54)	11 (50)	
1	8 (33)	5 (23)	
2	1 (4.2)	5 (23)	
3	1 (4.2)	0 (0)	
4	1 (4.2)	1 (4.5)	
Chronic GVHD, n (%)			0.459
No	18 (82)	13 (65)	
Limited	1 (4.5)	3 (15)	
Extensive	3 (14)	4 (20)	
CMV reactivation, n (%)	15 (63)	18 (82)	0.146
CMV disease, n (%)	2 (8.3)	2 (9.1)	1.000
EBV reactivation, n (%)	15 (63)	11 (50)	0.393
PTLD, n (%)	4 (17)	1 (4.5)	0.349
BKV reactivation, n (%)	19 (79)	18 (82)	1.000
Hemorrhagic cystitis, n (%)	3 (13)	9 (41)	0.028
Invasive fungal infection, n (%)	0 (0)	4 (18)	0.045
Sepsis/Pneumonia, n (%)	3 (13)	2 (9.1)	1.000
PIRCHE-II	High (≥ 32)	Low (< 32)	
Time to ANC 500 (days)	10 (8, 22)	11 (7, 15)	0.672
Time to PLT 200K (days)	20 (15, 180)	19 (15, 48)	0.446
Chimerism at d30)	100 (86.8, 100)	99.9 (65.1, 100)	0.599
Acute GVHD, n (%)			0.686
0	11 (46)	13 (59)	
1	7 (29)	6 (27)	
2	3 (13)	3 (14)	
3	1 (4.2)	0 (0)	
4	2 (8.3)	0 (0)	
Chronic GVHD, n (%)			0.605
No	15 (68)	16 (80)	
Limited	2 (9.1)	2 (10)	
Extensive	5 (23)	2 (10)	
CMV reactivation, n (%)	18 (75)	15 (68)	0.608
CMV disease, n (%)	3 (13)	1 (4.5)	0.609
EBV reactivation, n (%)	15 (63)	11 (50)	0.393
PTLD, n (%)	3 (13)	2 (9.1)	1.000
BKV reactivation, n (%)	20 (83)	17 (77)	0.718
Hemorrhagic cystitis, n (%)	6 (25)	6 (27)	0.861
Invasive fungal infection, n (%)	1 (4.2)	3 (14)	0.336
Sepsis/Pneumonia, n (%)	2 (8.3)	3 (14)	0.659

* High and Low PIRCHE Scores were determined based on the median of the respective score distributions in our cohort.

ANC, absolute neutrophil count; GVHD, graft-versus-host disease; CMV, cytomegalovirus; EBV, Epstein-Barr virus; PTLD, post-transplant lymphoproliferative disorder; BKV, BK virus.

For PIRCHE-II, there were no significant differences in both hematologic outcomes and other complications. Chronic GVHD was slightly more prevalent in the high-score group (32%) compared to the low-score group (20%), though this was not statistically significant (*P* = 0.605).

### Survival outcomes and association with PIRCHE scores

We conducted both univariable and multivariable Cox proportional hazards regression analyses to assess the impact of PIRCHE scores on survival outcomes, treating these scores as continuous variables and applying a log transformation.

**Figure 1:**
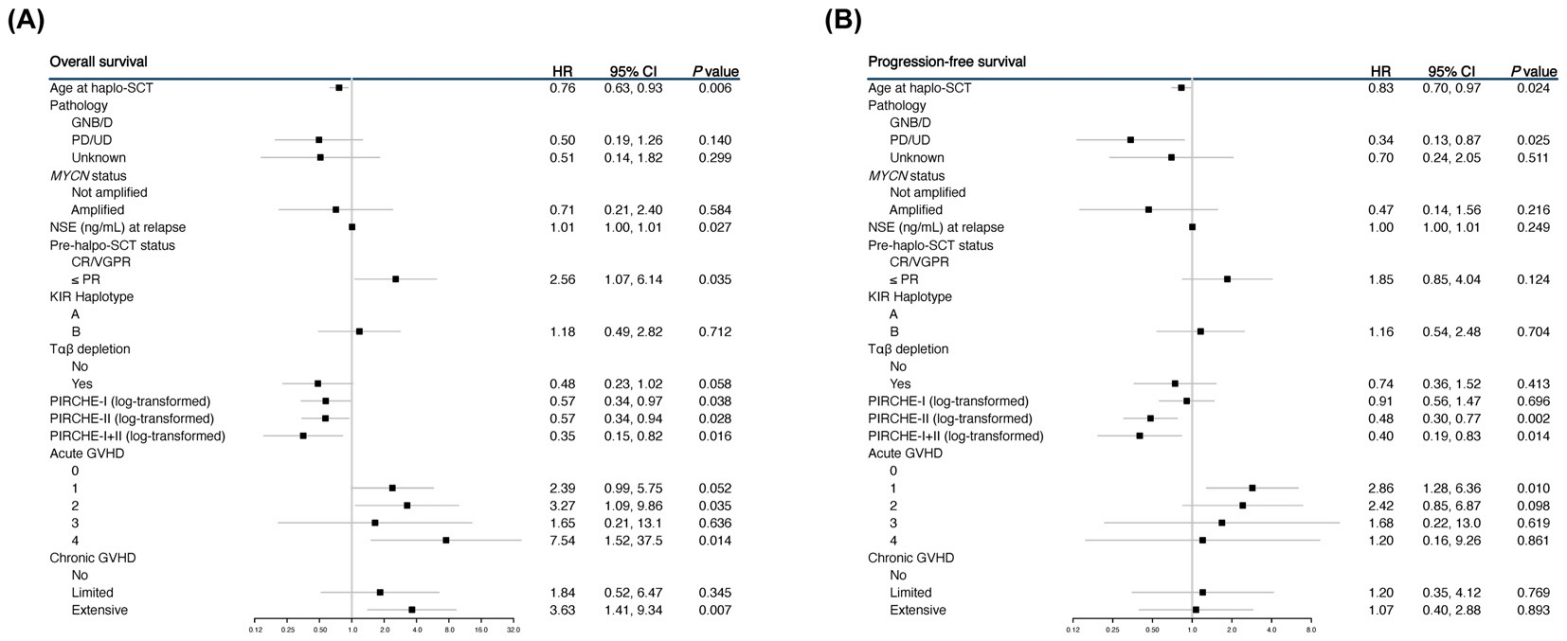
Forest plots showing the univariable analyses for overall survival (OS) **(A)** and progression-free survival (PFS) **(B)**.

In the univariable analysis, several significant associations between higher PIRCHE scores and survival outcomes were identified. For PIRCHE-I, higher scores were significantly associated with a lower risk of death (HR 0.57, 95% CI 0.34-0.97, *P* = 0.038) (**Figure 1A**). The high PIRCHE-II scores were significantly associated with both better OS (HR 0.57, 95% CI 0.34-0.94, *P* = 0.028) (**Figure 1A**) and reduced progression (HR 0.48, 95% CI 0.30-0.77, *P* = 0.002) (**Figure 1B**). The combined effect of higher PIRCHE I+II scores showed a more significant association with improved OS (HR 0.35, 95% CI 0.15-0.82, *P* = 0.016) (**Figure 1A**).

**Figure 2:**
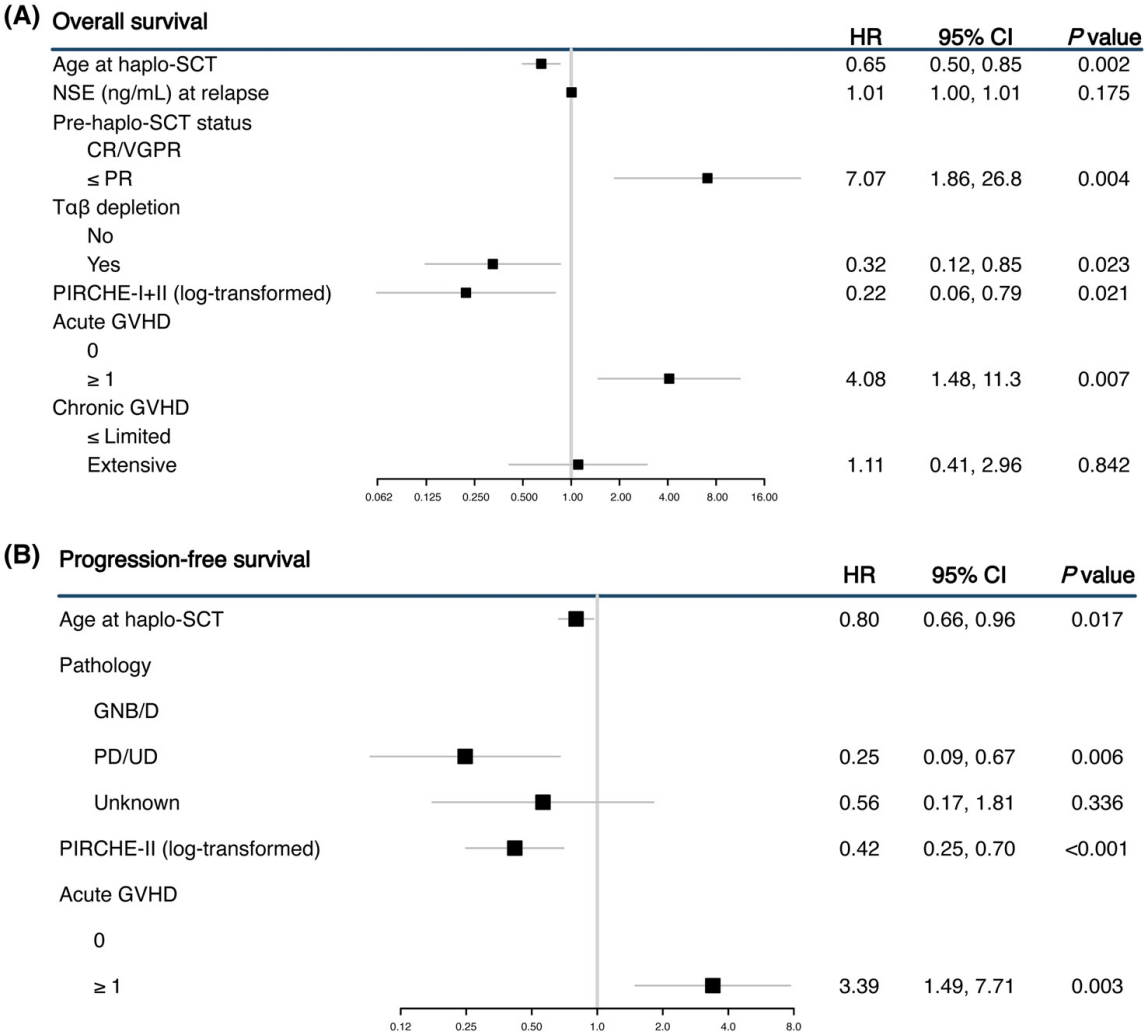
Forest plots showing the multivariable analyses for overall survival (OS) **(A)** and progression-free survival (PFS) **(B)**. The multivariable analysis was limited to variables that showed significance in the univariable analysis.

The multivariable analysis included variables with *P* value < 0.1 from the univariable analysis (**Figure 2**). For OS, higher log-transformed PIRCHE scores remained significantly associated with better outcomes. Specifically, the log-transformed combined PIRCHE score had a hazard ratio of 0.22 (95% CI 0.06-0.79, *P* = 0.021). Additionally, older age at haplo-SCT and receiving Tαβ depletion was significantly associated with better OS (HR 0.32, 95% CI 0.12-0.85, *P* = 0.023), while poorer response to treatment before haplo-SCT (≤ PR) was associated with worse OS (HR 7.07, 95% CI 1.86-26.8, *P* = 0.004). For progression, the multivariable analysis showed that higher PIRCHE-II scores were most significantly associated with a reduced risk (HR 0.42, 95% CI 0.25-0.70, *P* < 0.001).

## Discussion

Haplo-SCT preceded by HD-MIBG treatment is a feasible option for children with high-risk neuroblastoma who experienced relapse/progression after HDCT/auto-SCT (16, 21, 22). Although outcomes are not yet satisfactory, efforts should be made to improve survival, and selection of an optimal donor is an essential part of these efforts. In this study, we investigated the validity of the PIRCHE model in haploidentical donor selection in pediatric patients with relapsed/progressed neuroblastoma. A higher PIRCHE-I score was associated with faster hematologic recovery, less infection and better OS, while a higher PIRCHE-II score was associated with better PFS and OS in children with relapsed neuroblastoma who underwent haplo-SCT. Altogether, these findings support integration of the PIRCHE scoring system into the donor selection process for haplo-SCT in children with neuroblastoma.

Our study adds to the growing body of literature on the relationship between PIRCHE scores and transplant outcomes. Prior studies, primarily conducted in adults with hematologic diseases, reported conflicting results regarding PIRCHE scores and outcomes such as GVHD, non-relapse mortality and disease relapse (1, 8, 10, 23–27). These discrepancies likely arise from differences in patient populations, the transplantation protocols, HLA disparities, and clinical end points. In our pediatric neuroblastoma cohort, we observed distinct associations between PIRCHE scores and transplant outcomes, highlighting the context dependent nature of these metrics.

Patients with higher PIRCHE-I scores experienced faster platelet recovery and a reduced incidence of hemorrhagic cystitis and fungal infections, suggesting that HLA class I mismatches enhance hematologic recovery and immune reconstitution primarily through cytotoxic CD8+ T cells. Since HLA class I molecules are predominantly recognized by CD8+ T cells, these T cells may contribute to hematologic recovery by targeting and eliminating residual host hematopoietic cells, thereby promoting engraftment. Additionally, the enhanced immune recognition mediated by CD8+ T cells may improve immunity, reducing the risk of infections.

In contrast, higher PIRCHE-II scores, representing HLA class II mismatches, were not significantly associated with non-survival outcomes but were correlated with improved PFS and OS. These findings suggest that class II molecules, recognized by CD4+ helper T cells, likely contribute to GVT effects, which are critical for reducing relapse rates in neuroblastoma. This finding aligns with previous reports that HLA class II mismatches can enhance survival and GVT effects in haplo-SCT without significantly increasing in non-relapse mortality (28, 29). Specifically, mismatches at HLA-DR and HLA-DQ have been linked to enhanced survival and GVT effects. These results reinforce our findings that HLA class II mismatches had a more prominent role in GVT effects than class I mismatches in this setting.

In solid organ transplantation, higher PIRCHE-II scores may trigger an aggressive host-versus-graft immune response, potentially leading to graft rejection (30). However, in hematopoietic SCT, host-versus-graft reactions are minimized by conditioning regimens designed to suppress host immunity, emphasizing graft-versus-host interactions. The observed GVT effects, mediated by HLA class II mismatches, likely contributed to improved outcomes in our cohort.

Although greater HLA mismatches have been associated with increased GVHD risk in some studies (26), we did not demonstrate an association between PIRCHE score and GVHD incidence. A possible explanation is that the use of anti-thymocyte globulin with or without ex-vivo T cell depletion and reduced intensity conditioning could have weakened the impact of donor-recipient HLA epitope mismatches on GVHD. The absence of GVHD association in our study suggests the potential utility of PIRCHE scores for guiding donor selection without increasing GVHD risk. However, these results should be interpreted with caution, as the outcomes observed in this study may be influenced by the unique characteristics of our cohort and may not be generalizable to all patient populations.

While earlier investigations have yielded mixed results regarding PIRCHE scores and survival outcomes (8, 23, 25), our findings in pediatric neuroblastoma underscore the unique predictive value of these scores. Unlike studies that focused on adult patients with hematologic diseases or included transplants with varying levels of HLA disparity (10, 25–27), our study found significant associations between both PIRCHE-I and PIRCHE-II scores and OS. These results may be attributed to the lower incidence of infectious complications with higher PIRCHE-I scores and lower risk of relapse/progression associated with higher PIRCHE-II scores.

Emerging approaches that combine PIRCHE scores with other analyses could further enhance predictions of transplant outcomes (31–33). For example, integrating PIRCHE scores with eplet mismatch analyses could improve outcome predictions by simulating both B-cell- and T-cell-mediated alloimmune responses. These strategies, already promising in solid organ transplantation, may also refine donor selection in hematopoietic SCT by predicting donor-specific antibody production, which impacts engraftment and infection risk (34). Novel epitope-based algorithms such as Snowflake (35), could offer additional opportunities to improve predictive accuracy and clinical outcomes.

This study has several limitations. The retrospective design and single-center nature may limit the generalizability of our findings. Additionally, the relatively small sample size may affect the statistical power of our analyses, particularly for less common outcomes. Changes in GVHD prophylaxis strategy during the study period also are a limitation of our study, leading to heterogeneity of the cohort. Furthermore, as haplo-SCT is not well-established for childhood solid tumors, including neuroblastoma, we were unable to confirm the observed associations when applying different conditioning regimens or GVHD prophylaxis approaches, such as post-transplant cyclophosphamide. This variability limits direct comparisons with other studies and emphasizes the need for standardized protocols in this context. Another notable limitation is the lack of data for HLA-DPB1 and DRB3/4/5 loci in most donor-recipient pairs. While allelic matching at HLA-A, -B, -C, and -DRB1 is considered the standard for unrelated HCT, increasing evidence suggests that matching at DPB1 and DRB3/4/5 loci also impacts transplant survival and GVHD risk. Recent studies indicate that mismatches at these loci, despite lower expression levels, can influence transplant outcomes (1, 8, 20, 36–38). Incorporating these loci into PIRCHE score calculations may improve the predictive accuracy of outcomes in future studies. Lastly, we acknowledge that the use of arbitrary dichotomization for stratifying patients based on PIRCHE scores may be another limitation. While this approach allowed for a balanced comparison, future studies may explore alternative methods to enhance statistical robustness.

Despite these limitations, our cohort is strikingly different from those in other cohorts in that they are children with relapsed neuroblastoma who underwent haplo-SCT from a parent donor and received relatively uniform treatments compared to other cohorts. This unique patient population underscores the potential value of the PIRCHE scoring system in optimizing donor selection and improving outcomes.

In conclusion, our study demonstrates that higher PIRCHE-I and PIRCHE-II scores are associated with improved survival outcomes and reduced post-transplant complications in children with relapsed neuroblastoma undergoing haplo-SCT. These findings support the incorporation of PIRCHE scoring into pre-transplant risk assessment and patient stratification to optimize transplant outcomes. Further research should aim to validate these findings, explore the underlying mechanisms, and evaluate broader clinical applications of PIRCHE scores in the haplo-SCT setting.

## Data Availability

The original contributions presented in the study are included in the article/supplementary material. Further inquiries can be directed to the corresponding authors.
