# Corrigendum: Predicted indirectly recognizable HLA epitopes scores and clinical outcomes after haploidentical stem cell transplantation in pediatric patients with relapsed neuroblastoma

**DOI:** 10.3389/fimmu.2025.1565304

**Published:** 2025-02-04

**Authors:** Eun Seop Seo, In Hwa Jeong, Hee Young Ju, Ju Kyung Hyun, Ji Won Lee, Keon Hee Yoo, Won Young Heo, Ki Woong Sung, Hee Won Cho, Eun-Suk Kang

**Affiliations:** ^1^ Department of Pediatrics, Samsung Medical Center, Sungkyunkwan University School of Medicine, Seoul, Republic of Korea; ^2^ Department of Digital Health, Samsung Advanced Institute for Health Sciences & Technology (SAIHST), Sungkyunkwan University, Seoul, Republic of Korea; ^3^ Department of Laboratory Medicine and Genetics, Samsung Medical Center, Sungkyunkwan University School of Medicine, Seoul, Republic of Korea; ^4^ Department of Laboratory Medicine, Dong-A University Hospital, Dong-A University College of Medicine, Busan, Republic of Korea

**Keywords:** neuroblastoma, PIRCHE, haploidentical hematopoietic stem cell transplantation, relapse/refractory, HLA mismatch

In the published article, there was an error in the **Funding** statement. The original funding statement is as follows: “The author(s) declare financial support was received for the research, authorship, and/or publication of this article. This work was supported by the National Research Foundation of Korea (NRF) grant, funded by the Korean government (MSIT) (2017R1A2B4008178). Additionally, this research was supported by a grant from the Korea Health Technology R&D Project through the Korea Health Industry Development Institute (KHIDI), funded by the Ministry of Health and Welfare, Republic of Korea (grant number: HA15C0007).”

The correct **Funding** statement appears below.

“The author(s) declare financial support was received for the research, authorship, and/or publication of this article. This work was supported by the National Research Foundation of Korea (NRF) grant, funded by the Korean government (MSIT) (2017R1A2B4008178, 2022R1F1A1075238). Additionally, this research was supported by a grant from the Korea Health Technology R&D Project through the Korea Health Industry Development Institute (KHIDI), funded by the Ministry of Health and Welfare, Republic of Korea (grant number: HA15C0007).”

The authors apologize for this error and state that this does not change the scientific conclusions of the article in any way. The original article has been updated.

